# Genotypic-specific variance in *Caenorhabditis elegans* lifetime fecundity

**DOI:** 10.1002/ece3.1057

**Published:** 2014-04-24

**Authors:** S Anaid Diaz, Mark Viney

**Affiliations:** School of Biological Sciences, University of BristolWoodland Road, Bristol, BS8 1UG, UK

**Keywords:** *Caenorhabditis elegans*, fitness, isogenic, lifetime fecundity, reproduction, variance

## Abstract

Organisms live in heterogeneous environments, so strategies that maximze fitness in such environments will evolve. Variation in traits is important because it is the raw material on which natural selection acts during evolution. Phenotypic variation is usually thought to be due to genetic variation and/or environmentally induced effects. Therefore, genetically identical individuals in a constant environment should have invariant traits. Clearly, genetically identical individuals do differ phenotypically, usually thought to be due to stochastic processes. It is now becoming clear, especially from studies of unicellular species, that phenotypic variance among genetically identical individuals in a constant environment can be genetically controlled and that therefore, in principle, this can be subject to selection. However, there has been little investigation of these phenomena in multicellular species. Here, we have studied the mean lifetime fecundity (thus a trait likely to be relevant to reproductive success), and variance in lifetime fecundity, in recently-wild isolates of the model nematode *Caenorhabditis elegans*. We found that these genotypes differed in their variance in lifetime fecundity: some had high variance in fecundity, others very low variance. We find that this variance in lifetime fecundity was negatively related to the mean lifetime fecundity of the lines, and that the variance of the lines was positively correlated between environments. We suggest that the variance in lifetime fecundity may be a bet-hedging strategy used by this species.

## Introduction

Evolutionary success is achieved by maximizing fitness in spatially and temporally variable environments. Key to this is phenotypic variation, which can be due to genetic differences among individuals and/or due to environmentally induced effects, *i.e*. phenotypic plasticity. However, in a constant environment genetically identical individuals are assumed to be phenotypically constant. Phenotypic differences among such individuals are usually attributed to stochastic events that would generally result in equal degrees of phenotypic variance among different genotypes. Importantly, implicit in the assumption of stochasticity underlying phenotypic variance among genetically identical individuals is that the phenotypic variance is not genetically controlled, not subject to selection, and thus not adaptive.

Contrary to this assumption, phenotypic variance in isogenic populations in constant environments is observed (Viney and Reece 2013). Beyond stochastically generated differences, microenvironmental differences among individuals, such that each individual is in effect in its own microenvironment, could also cause such phenotypic variance. However, the relative roles of stochasticity and microenvironmental differences in causing phenotypic variance are not known.

One adaptive explanation of the phenomenon of phenotypic variance in isogenic populations is the evolution of bet-hedging or risk-spreading strategies, which makes the link between environmental variability, fitness variance and mean fitness (Gillespie 1974). Bet-hedging strategies are those that minimize fitness variation across generations in the face of environmental variation. Thus, when comparing phenotypically invariant and variant genotypes, an invariant genotype would have a certain fitness in one environment to which it was adapted, but a potentially steep fitness decline outside of that environment. In contrast, a phenotypically variant genotype could have a comparatively greater fitness in heterogeneous environments because its fitness would not decline so quickly as the environment changed. Therefore, phenotypic variance increases the fit phenotypic space of a genotype. The repeated observation of microorganismal phenotypic variance within a clone has been interpreted as maximizing fitness in variable environments (Dubnau and Losick 2006). Other, related approaches to understanding phenotypic variance center around the concept of canlization of phenotypic traits, defined as the robustness of phenotypes to perturbation (Flatt 2005). Despite much working demonstrating different degrees of canalization of different phenotypes, the significance of different degrees of phenotypic variation among genotypes has largely not been considered (Gibson and Dworkin 2004).

Theoretical studies have shown how selection can change phenotypic variance (*e.g*. Gillespie 1974; Hill and Zhang 2004) and mechanistically phenotypic variance can be genetically controlled, changing the phenotypic robustness of a population (Hermisson and Wagner 2004). Much of the empirical work describing the molecular and genetic mechanism of phenotypic variance of genetically identical individuals in constant environments has focused on unicellular systems (Davidson and Surette 2008; Viney and Reece 2013). In many of these systems there is variability in gene expression (Maamar et al. 2007), often affecting whole gene networks, which if manifest as phenotypic variance may allow selection to act on trait variance (Viney and Reece 2013). The effect of mutations on trait variance (as well as on trait values) has been observed in multicellular organisms too (Dunn and Fraser 1958). For example, wild-type mice have 18–19 whiskers, but the *Tabby* mutation results in fewer whiskers (mean 12) and a greater variance in whisker number (range 8–16) (Dunn and Fraser 1958). There are also empirical data showing the genetic control of phenotypic variance among genetically identical individuals in a constant environment. For example, *Caenorhabditis elegans* mutations that affect the developmental cell lineage (which in wild-type is essentially invariant among individuals) are incompletely penetrant, *i.e*. genetically identical individuals vary phenotypically (Horvitz and Sulston 1980; Braendle and Félix 2008; Braendle et al. 2010). In this system, there is a molecular understanding of how incomplete penetrance of *C. elegans* phenotypes occurs in certain traits. The specification of gut cells is controlled by a small transcription network and variation in gene expression near a threshold alters the phenotype of individuals, thereby altering penetrance (Raj et al. 2010). Trait variance is of increasing interest in animal breeding settings where low trait variance can be desirable as a means to maximize production gains and agricultural efficiency. The study of trait variance has extended to studies in humans, for example looking at the genetic control of variability in body mass index (Yang et al. 2012).

Laboratory studies of unicellular species show that among genetically identically individuals there is phenotypic variation that can be genetically controlled and that can be adaptive (Viney and Reece 2013). For multicellular species, though, there has been much less investigation of these phenomena. Also, empirical data supporting the idea that phenotypic variance amongst genetically identical individuals in a constant environment may contribute to reproductive success is limited. Here, we quantified the phenotypic variance among genetically identical individuals in the model nematode system *C. elegans*. This system allows accurate quantitation of many aspects of individuals' phenotypes, crucially including lifetime fecundity. We have therefore examined the lifetime fecundity of *C. elegans* and, more particularly, how this varies among individuals. Specifically, we asked for an inbred line of *C. elegans* in one environment how much phenotypic variance in lifetime fecundity there was among individuals. We compared 20 recently wild isolates of *C. elegans* in this way and found that the among individual variation in lifetime fecundity of each line varied significantly among the *C. elegans* isolates. This means that the *C. elegans* genotype – a genetic effect – affects phenotypic variance. We then repeated this but using different food environments, which tests how the environment itself affects this phenotypic variance. These different environments changed the phenotypic variance of the lines. Also, within each food environment we found that there was a negative relationship between the lifetime fecundity and the among individual variance in lifetime fecundity. Furthermore, we found that these variances in lifetime fecundity correlated positively across the two food environments. We suggest that these results may show that these *C. elegans* isolates display a gradient of risk-spreading strategies.

## Materials and Methods

### Worms and food

We used 20 recently wild (see Appendix 1) *C. elegans* isolates and the standard N2 wild-type. For each isolate, one isogenic line was made by single-worm inbreeding for at least 10 generations (inbreeding coefficient > 0.9) and cryopreserved; hereafter these isogenic lines are referred to as lines. Each experiment used a new cryopreserved stock of the isogenic line. We used two food sources, *Escherichia coli* OP50 and *Bacillus pumilus* B215 (see Appendix 1).

### Assays

Lifetime fecundity was the total number of viable progeny produced by individual hermaphrodites grown with excess food at 19°C. To measure this, arrested first stage larvae were generated by hypochlorite treatment from which synchronized L1s hatched, and after 24 hours 10 individuals of each line were individually introduced to food (day 0), with five individuals for each food source. Worms grew into adult hermaphrodites, which were transferred every other day to NGM fresh plates (Hope 1999) for their reproductive life. Egg-containing plates (from which adult hermaphrodites had been removed) were maintained for 48 hours, when the number of viable progeny were counted. We conducted the observations in three experimental blocks, and all lines and both food sources were included in each block; thus, 3 blocks × 21 lines × 5 worm replicates = 315 individual observations per food source (see Appendix 2: Tables A2 and A3). We found no differences among the blocks (see below) and so the block data for each food source was pooled.

### Statistical models and analyses

Our objectives were to analyse how the (i) mean lifetime fecundity (*LF*) and (ii) the variance in *LF* varied among the *C. elegans* lines and between the two food sources. We used a Bayesian modelling approach, using a hierarchical linear model, to compare different distributions of the mean *LF* and the variance in *LF*. From this we then concluded how the *LF* and variance in *LF* varied among the lines and between the two food sources.

Our initial Model 1 was:


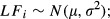
(Model 1)

where *LF*_*i*_ is the *LF* of the *i*th individual worm of all of the observations that we made, *N* denotes a normal distribution, *μ* is the mean *LF* across the *i* observations (*i* = 315), and *σ*^2^ is the variance across *LF* observations. *μ* has an *N*(0, 1*e*10) prior distribution and the precisions *τ* = 1/*σ*^2^ has *G* (0.001, 0.001) prior (so the gamma distribution has extremely large standard deviations). The relevant computer code for these models is available in Appendix 3.

The worms were grown in two different food environments so we applied Model 1 to the *LF* data from these two food environments separately. This showed that the mean *LF* of the lines differed significantly between the two food environments, shown by the non overlap of the credible intervals (CI) of the mean *LF*; mean *LF* on *B. pumilus* 98.76, CI 91.31–106.3; mean *LF* on *E. coli* 244.9, CI 236.3–253.6. We therefore analysed the *LF* from the two food environments separately.

We next extended Model 1 to make Model 2:


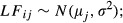
(Model 2)

which models the data such that each *C. elegans* line, *j* (*j* = 1…21), has its own mean *LF*, denoted by *μ*_*j*_, but all lines have the same variance in *LF*, denoted by *σ*^2^, referred to as the residual variance. This model has a *μ*_*j*_ with an *N (θ, σ*^2^_among_*)* prior distribution, where the hyperparameter *θ* has a *N* (0, 1*e*10) hyperprior, and the precisions *τ*_among_ = 1/*σ*^2^_among_ and *τ* = 1/*σ*^2^ both have *G* (0.001, 0.001) priors. *θ* represents the mean *LF* among lines, and *σ*^2^_among_ the among line variance which quantifies how the mean *LF* varies among the lines. The relative contributions (interclass correlation coefficient) that each component contributes to the total variance are, for example, *σ*^2^_among_/(*σ*^2^_among_ + *σ*^2^); these are presented expressed as percentages. If Model 2 was a better model than Model 1 then we concluded that the *C. elegans* lines have different *LF*. We describe later in this section how we compared models.

We then extended Model 2 to make Model 3:


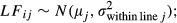
(Model 3)

which models the data such that each *C. elegans* line, *j*, also has its own variance in *LF*, denoted by *σ*^2^_within line *j*_. This model had with an *N (θ, σ*^2^) prior distribution, a *N* (0, 1*e*10) hyperprior and the precisions are *τ*_within line *j*_ = 1/*σ*^2^_within line *j*_ and *τ* = 1/*σ*^2^, both have *G* (0.001, 0.001) priors. If Model 3 was a better model than Model 2 then we concluded that the *C. elegans* lines have different variances in *LF*.

We used the same approach to investigate whether there were differences in *LF* among the different experimental blocks. We did this by extending Model 3, making Model 4:


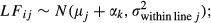
(Model 4)

which accounts for the three experimental blocks by including the term *α*_*k*_, where *k* is the number of experimental blocks (*k* = 3), with a *N* (0, 1) prior. When we used this model it was unable to converge on a solution for this coefficient (*i.e*. an identifiability issue, Spiegelhalter et al. 2002). This suggests either that the block effect was negligible or that the dataset was too small to allow for an analysis of block effects. We therefore reduced the number of terms in the model to test for an effect of experimental block by extending Model 2 to include the experimental block parameter, *α*_*k*_, making Model 5:


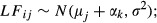
(Model 5)

If Model 5 was a better model than Model 2 we concluded that there are significant effects of the three experimental blocks.

We compared models by calculating the differences in deviance information criterion (ΔDIC) between two models. To choose the best model, a ΔDIC of less than 2 was taken to indicate substantial support for the simpler model, a ΔDIC of between 4 and 7 was taken to indicate considerably less support for the simpler model, and a ΔDIC of greater than 10 was taken to indicate essentially no support for the simpler model (Spiegelhalter et al. 2002).

We constructed the models using WinBUGS, which is a software package that uses Markov chain Monte Carlo (MCMC) methods to fit Bayesian statistical models (Lunn et al. 2000); this code is available in Appendix 3; Table A1 presents the DIC values of models 1 – 3. For the results, the reported parameters are after 10,000 iterations, then followed by a further 10,000 updates for the parameters. This produced a Monte Carlo error of less than 5% of the posterior standard deviation. Throughout the variance is described as standard deviations unless otherwise stated; estimates are presented as the mean with its respective 2.5 and 97.5% CI.

## Results

We firstly consider whether there was any effect of the experimental block. There was no effect of experimental block on the mean *LF* of the lines, shown by the ΔDIC, on the *B. pumilus* food source (ΔDIC _Model 3 – Model 4_ = 0.11; ΔDIC _Model 2 – Model 5_ = −0.1) or on the *E. coli* food source (ΔDIC _Model 3 – Model 4_ = 0.74; ΔDIC _Model 2 – Model 5_ = 0.53), and so data from all three blocks were analysed together.

The mean *LF* varied between the two food sources with those worms eating *B. pumilius* having approximately two-fifths of the progeny of worms eating *E. coli* (see Appendix 2 Table A1). The results from Model 1 showed that the mean *LF* of the lines differed significantly between the two food environments, shown by the nonoverlap of the credible intervals (CI) of the mean *LF*; mean *LF* on *B. pumilus* 98.76, CI 91.31–106.3; mean *LF* on *E. coli* 244.9, CI 236.3–253.6.

**Figure 1:**
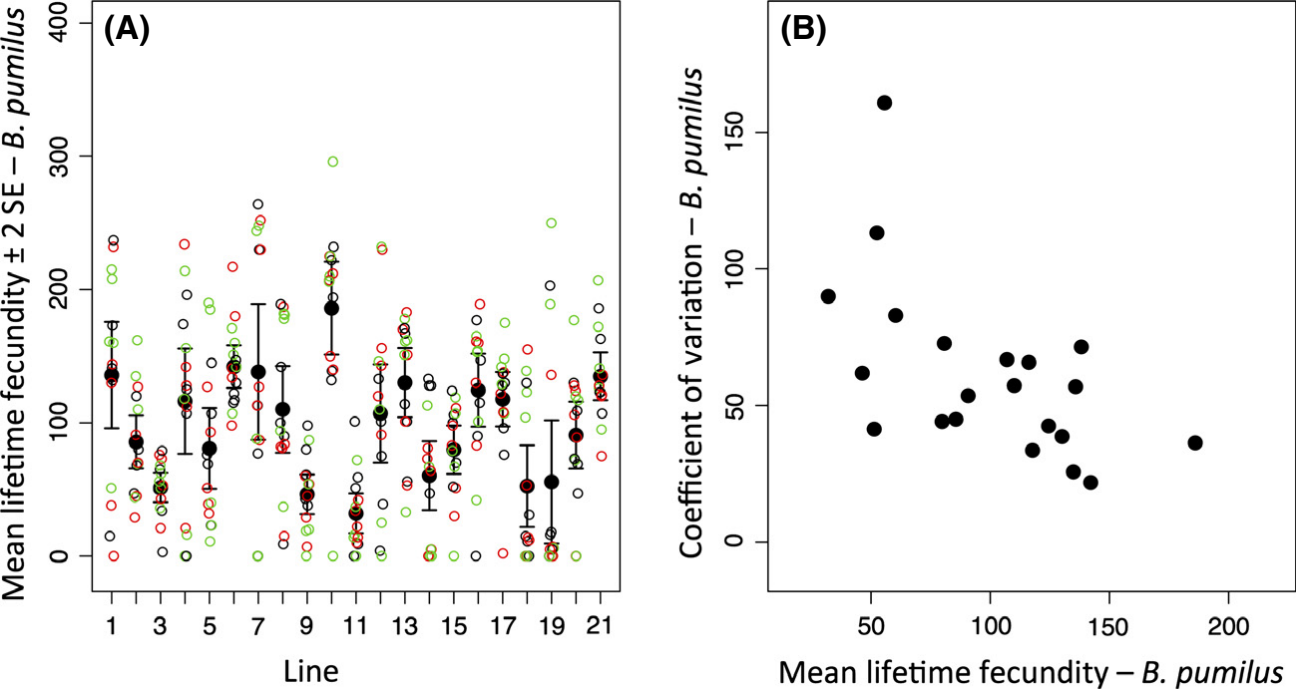
(A) Mean lifetime fecundity (± 2SE) of *C. elegans* lines fed *B. pumilus* B215. 1 = N2, 2 = CB4853, 3 = JU1400, 4 = JU1401, 5 = JU1409, 6 = JU1410, 7 = JU1411, 8 = JU1416, 9 = JU1442, 10 = JU1494, 11 = JU262, 12 = JU319, 13 = JU345, 14 = JU362, 15 = JU393, 16 = JU400, 17 = MY1, 18 = MY16, 19 = MY2, 20 = PX174 and 21 = PX179. Open circles represent the individual observations, colored by block (black, red and green, for block one, two and three respectively). (B) Relationship between a line's mean lifetime fecundity and its coefficient of variation (CV). A line's mean and CV of lifetime fecundity are estimates across blocks' pooled data.

Considering the *LF* of worms when fed *B. pumilius*, the mean *LF* of the isogenic lines ranged from 35–167 [Fig. 1A; Appendix 2 Table A2(A)]. The lines differed in their *LF* in this food environment (difference in mean *LF* among lines, ΔDIC_Model 1 – Model 2_ = 86.7, Table 1, Fig. 1A). Analysis of variance components showed that the among line variance was greater than the residual variance (108.4 [79.8–149.6 CI] and 57.5 [52.9–62.3 CI], *σ*_among_ and *σ*, respectively, Model 2), suggesting that *c*. 65% of the observed variance in *LF* was among lines, and the remainder the residual variance.

**Table 1:** DIC results of the models for *B. pumilus* B215 and *E. coli* OP50. The results show Dbar, Dhat and pD. Dbar is −2 times the sample average of the log-likelihoods; Dhat is −2 times the log-likelihood evaluated at the posterior mean of the parameters; pD, calculated as Dbar − Dhat, is the effective number of parameters in the model. The most parsimonious model according to ΔDIC is shown in bold

	Dbar	Dhat	pD	DIC
*B. pumilus*				
Model				
Model 1	3551.98	3549.9	2.0	3553.9
Model 2	3445.4	3423.7	21.7	3467.2
**Model 3**	**3373.2**	**3332.3**	**40.9**	**3414.1**
*E. coli*				
Model				
Model 1	3643.2	3641.2	1.9	3645.2
Model 2	3531.5	3509.5	22.0	3553.5
**Model 3**	**3447.1**	**3406.3**	**40.9**	**3488.0**

**Figure A1:**
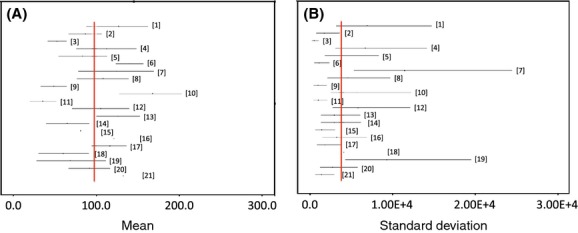
Results of Model 3 (see main text, Table 1) showing the posterior distributions of (A) mean (*μ*_*j*_) and (B) standard deviation (σ_within line *j*_) ± CI (Credible Intervals, from Appendix 2 Table 2) of *LF* of lines fed *B. pumilus* B215. The *C. elegans* line number is shown is shown in brackets (Appendix 1). The vertical lines represent the global mean of the posterior means and standard deviations among lines, shown in Appendix 2 Table 2.

We then further explored the differences among lines by describing the variation within each line; specifically, we wanted to test whether including an extra parameter to describe the within line variance in *LF* for each line improved the fit of the model. The analysis showed that Model 3, which included the within line variance, was the preferred model (ΔDIC_Model 2 – Model 3_ = 53.1, Table 1), consistent with the within line variance in *LF* varying significantly among the *C. elegans* lines. The within line variance in *LF* ranged between 525.8 and 11310.0 [*σ*_within line_ line 3 and 7, respectively, Fig. 1A; Appendix 2 Table A1(A) and Fig. A1]. Line 7 showed a variance 21 times higher than that of line 3. Therefore, we conclude that in the *B*. *pumilus* food source there were significant differences in the mean *LF* among lines, and in the within line variance in *LF*. We also found that the lines' coefficient of variation (CV) and mean lifetime fecundity were correlated (Spearman coefficient *r* = −0.59, *P* < 0.01) (Fig. 1B). The lines did not differ in their schedule of reproduction, all commenced reproduction on day 2 and had completed it by day 8.

**Figure 2:**
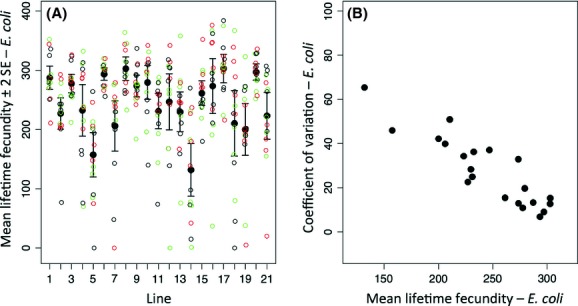
(A) Mean lifetime fecundity (± 2SE) and (B) the relationship between mean and CV of lifetime fecundity of *C. elegans* lines fed *E. coli* OP50; isogenic lines as Figure 1.

**Figure A2:**
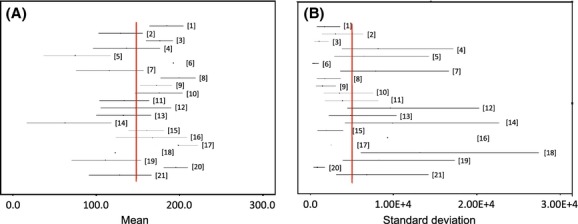
Results of Model 3 (see main text, Table 2) showing the posterior distributions of each (A) mean (*μ*_*j*_) and (B) standard deviation (σ^2^_within line *j*_) ± CI of *LF* of lines fed *E. coli* OP50. The *C. elegans* line number is shown is shown in brackets (Appendix 1). The vertical lines represent the global mean of the posterior means and standard deviations among lines, shown in Appendix 2 Table 2.

The *E. coli* food environment was significantly better than the *B. pumilus* environment, with the lines producing an average of 151 more offspring (Fig. 2A). In this better environment the mean *LF* [range 162–299, Fig. 2; Appendix 2 Table A1(B)] was different among the lines (ΔDIC_Model 1 – Model 2_ = 91.7, Table 1). The analysis of variance components showed that the among line variance was greater than the residual variance (256.4 [189.8–251.2 CI] and 65.8 [60.7–71.5 CI], *σ*_among_ and *σ*, respectively, Model 2), suggesting that *c*. 80% of the observed variance in *LF* was among lines, and the remainder the residual variance. Also, Model 3, that included the within line variance, was the preferred model (ΔDIC_Model 2 – Model 3_ = 65.5, Table 1), again consistent with the within line variance in *LF* varying among the *C. elegans* lines. For example, line 6 showed the lowest variance (*σ*_within line_ = 473.6 [218–1019 95% CI]; Appendix 2 Table A1(B) and Fig. A2), whereas line 18 showed a *c*. 28 times greater variance (*σ*_within line_ = 13180.0 [6076–27480 CI]; Appendix 2 Table A1(B) and Fig. A2). Therefore, similarly to the *B. pumilus* food environment, we conclude that in the *E*. *coli* food environment there were significant differences in the mean *LF* among lines and in the within line variance in *LF*.

In the *E. coli* environment there was a significant negative relationship between the mean and CV *LF* of the lines (*r* = −0.88, *P* < 0.001) (Fig. 2B). Reproduction occurred earlier on the *E. coli* food source (*E. coli* and *B. pumilus* maximum daily fecundity on day 2 and 4 respectively).

**Figure 3:**
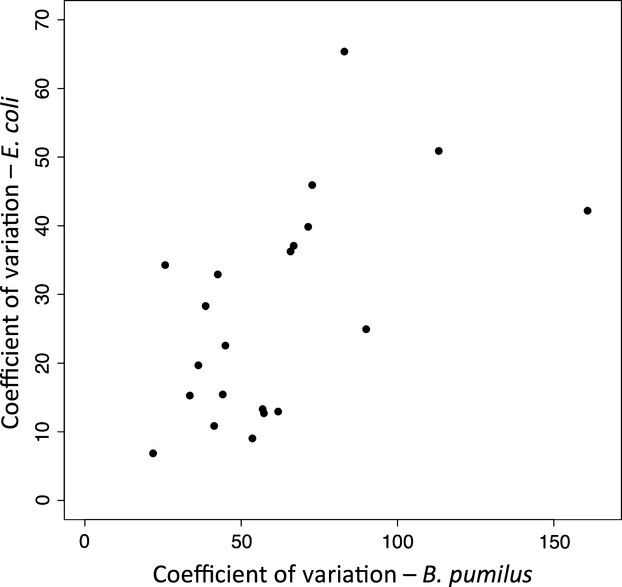
The relationship between the CV of lifetime fecundity of the *C. elegans* lines fed *B. pumilus* and *E. coli*.

**Figure A3:**
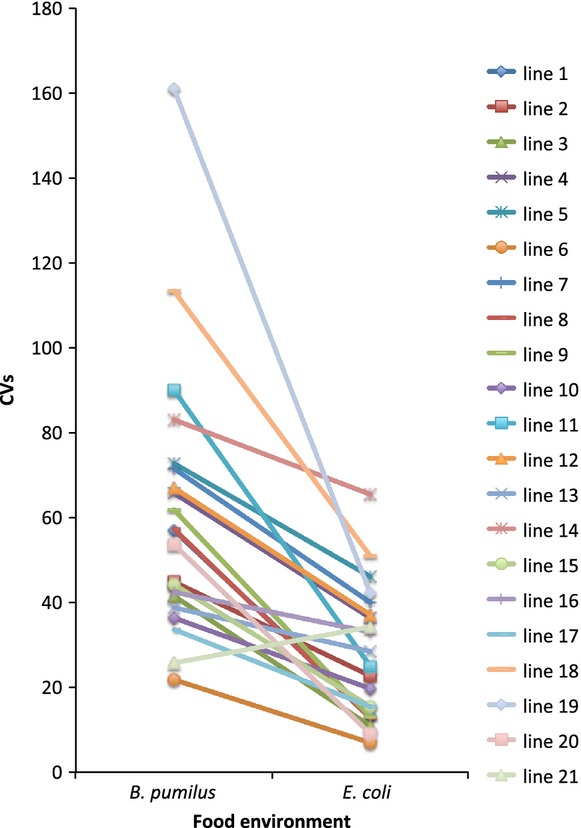
The CV in *LF* between the two food environments for each *C. elegans* line.

The within line variance in *LF* was greater in the poorer quality *B. pumilus* environment (shown by the CV values in each environment) showing that within line variance in *LF* is phenotypically plastic (Appendix 2 Fig. A3). There was a relationship in the CVs of the lines in the two food environments (*r* = 0.62, *P* = 0.002) (Fig. 3), but there was no such relationship between the *LF* of the lines (*r* = 0.29, *P* = 0.20).

## Discussion

Our results show that variance in lifetime fecundity within an isogenic line differs among *C. elegans* isogenic lines, thus suggesting there is a genetic effect on phenotypic variance. Specifically, we demonstrate that in the same environment, genetically identical individuals show different levels of among individual phenotypic variation depending on the genotype. There are genotypes with low phenotypic variation and others with high phenotypic variation. We therefore conclude that this phenotypic variance is a property of a genotype.

These results show that the assumption of the equality of phenotypic variance among genotypes is, in this case, false. Furthermore, that we observed that this phenotypic variance differed among recently wild genotypes suggests that this variance may be due to the genotypes' different evolutionary histories. Consistent with this notion is the fact that this phenotypic variance was plastic between the two food environments (Viney and Diaz 2012). Specifically, we find that the absolute degree of phenotypic variance of each of the lines changes as the food environment changes. However, each isogenic line's among-individual variation is similar in both of the two food environments as shown by the positive correlation between CVs. This suggests that although the environment affects the absolute level of variation (i.e. higher in the poorer quality environment) isogenic lines show a similar level of variation across environments.

We have also found that among the *C. elegans* iosgenic lines the variance in lifetime fecundity is negatively related to mean lifetime fecundity. Why might this pattern exist? We suggest that this may be evidence of different bet hedging strategies. Bet-hedging strategies maximize geometric mean fitness, but at a cost to arithmetic mean fitness. A detailed analysis of bet-hedging strategies has shown that this can be achieved (i) by reducing among individual variance in fitness across generations and (ii) by reducing the correlation in fitness among individuals within a generation (because this effectively decreases the variance in fitness across generations) with these approaches being considered conservative and diversified bet-hedging strategies respectively (Starrfelt and Kokko 2012). Conservative and diversified bet-hedging strategies are extremes along a continuum and both strategies can occur together because they are not mutually exclusive (Starrfelt and Kokko 2012).

To consider our observations with the perspective of bet-hedging strategies, let us assume that the variance in lifetime fecundity is a measure of the variance in the quality of the worms. With this assumption, the *C. elegans* lines then have different ranges of offspring quality. Considering the negative relationship that we observed between the mean and CV of lifetime fecundity, then among the 21 lines there are a gradient of phenotypes where the extremes are (i) lines of high fecundity, low among individual variance-in-quality through to (ii) lines of low fecundity, high among-individual variance-in-quality.

With this assumption, the low variance lines may be pursuing a conservative bet-hedging strategy, perhaps adapted to a stable, benign environment, such that the variance in fitness across generations is small. In contrast, the high variance lines may be using a diversified bet-hedging strategy, perhaps adapted to a spatially variable environment (Starrfelt and Kokko 2012) where within a generation there is variance among individuals in their fitness, but across generations the fitness variance is minimized, thereby maximizing geometric mean fitness.

The existence of high variance, low fecundity, lines might be puzzling because it would be seem to better for these high variance lines to have a high fecundity too. One reason for this may be that there is some limit or trade-off among individual progeny, such that if one individual offspring is high quality (observed here as having a high lifetime fecundity) other individuals have to be of low quality. This could be achieved in *C. elegans*, for example, by adult hermaphrodites differently allocating resources among offspring, which results in differences among individuals in their quality, which we observe as differences in lifetime fecundity.

There are caveats to interpreting the phenotypes of these lines as bet-hedging strategies. Thus, it is possible that in other environments the lines' lifetime fecundity and variance in lifetime fecundity will differ in other ways, and present other patterns (or no pattern at all), which might then argue against such a bet-hedging strategy existing in this species. More needs to be known about the natural environment of *C. elegans*, which is a subject of current work (Félix and Duveau 2012).

Our results can also be interpreted from the perspective of trait canalization (Flatt 2005). We have found that the lines differ in their variability in lifetime fecundity, which is consistent with some lines being strongly canalized for this trait whereas others are not (Baer 2008; Braendle et al. 2010). This view is then tacit about the adaptive value, if any, of this.

Other studies have shown how among individual, within genotype phenotypic variance can occur (*e.g*. Horvitz and Sulston 1980; Braendle and Félix 2008). A common understanding of the control of a trait is that it is tightly genetically controlled, and that relaxation of that control (for example by mutation) degrades the trait, seen as developmental errors or incomplete penetrance. Our results show that phenotypic variance in lifetime fecundity is a property of a genotype.

## Appendix 1: Worm and food strains

*C. elegans* recently wild isolates were obtained from two sources: JU1400, JU1401, JU1409, JU1410, JU1411, JU1416, JU1442, JU1494 from Marie-Anne Félix (Paris); CB4853, MY1, MY2, MY16, JU262, JU319, JU345, JU362, JU393, JU400, PX174, PX179 and the standard N2 wild-type was obtained from the *Caenorhabditis* Genetics Centre (CGC). These isolates were collected in France in 2001 (JU262), 2002 (JU319, JU345, JU362, JU393, JU400) or 2008 (JU1494); Spain in 2008 (JU1400, JU1401, JU1409, JU1410, JU1411, JU1416, JU1442); northwest Germany in 2002 (MY1, MY2, MY16); Oregon, USA in 2004 (PX174, PX179) and California, USA in 1974 (CB4853). This information is available at http://www.justbio.com/worms/index.php and http://www.cgc.cbs.umn.edu. The single-worm inbreeding was done on an *E. coli* OP50 food source. After inbreeding worms were cryopreserved (Hope 1999) and maintained at −80°C.

We wished to measure the lines' lifetime fecundity on two different food sources, *E. coli* OP50 and *B. pumilus* B215. The *E. coli* OP50 (the standard laboratory *C. elegans* food source) was obtained from CGC; the *Bacillus pumilus* B215 isolate which was obtained from Robbie Rae and Ralf Sommer (Tübingen) (Rae et al. 2010). We chose the *B. pumilus* B215 because pilot studies showed it to be not overtly pathological to the *C. elegans* lines and not to induce behaviors which would interfere with measurement of worms' lifetime fecundity. The *E. coli* OP50 and *B. pumilus* B215 were cultured as described previously (Hope 1999; Rae et al. 2010).

We observed minimal sterile hermaphrodites throughout out the experiment, specifically, on *E. coli* OP50 JU1409, JU1411, JU319, MY16 each had one; on *B. pumilus* B215 N2, JU1442, JU319, JU393, JU400 each had one, and JU1401, JU1411, JU1494, JU262, JU362, MY16, MY2, and PX174 between two and five. These data were included in the analyses. We use the following line codes to report the statistics: 1 = N2, 2 = CB4853, 3 = JU1400, 4 = JU1401, 5 = JU1409, 6 = JU1410, 7 = JU1411, 8 = JU1416, 9 = JU1442, 10 = JU1494, 11 = JU262, 12 = JU319, 13 = JU345, 14 = JU362, 15 = JU393, 16 = JU400, 17 = MY1, 18 = MY16, 19 = MY2, 20 = PX174, and 21 = PX179.

## Appendix 2

Model results: Table A1.

**Table A1:** Results of preferred Model 3 (see main text, Table 1). It includes the mean, SD and the 2.5 and 97.5% credible intervals (CI) of the posterior distribution for *θ* (theta), *μ* (mu), and σ_within line *j*_ (sigma.with.line) of each line for (A) *B. pumilus* B215 and (B) *E. coli* OP50. These results are of 30000 sampled values at 10001 post burn-in

Node	Mean	SD	2.5% CI	97.5% CI
(A) *B. pumilus*				
Theta	97.64	9.20	79.71	116.00
mu[1]	126.50	18.95	88.37	162.90
mu[2]	86.50	10.22	66.49	107.00
mu[3]	52.48	5.86	41.03	64.34
mu[4]	111.90	18.38	75.10	148.10
mu[5]	83.39	14.86	54.16	112.90
mu[6]	139.80	8.55	122.20	156.20
mu[7]	124.60	22.56	79.17	168.40
mu[8]	107.80	15.83	75.98	138.60
mu[9]	48.57	7.96	33.23	64.78
mu[10]	167.30	19.26	126.30	202.30
mu[11]	35.16	8.09	19.78	51.80
mu[12]	105.00	17.32	70.98	139.40
mu[13]	126.10	13.23	99.02	151.90
mu[14]	65.02	13.14	39.53	92.04
mu[15]	80.99	9.42	62.40	99.70
mu[16]	120.80	13.78	92.75	147.60
mu[17]	116.10	10.51	94.86	136.80
mu[18]	59.88	15.37	30.43	91.21
mu[19]	68.13	21.11	27.12	110.70
mu[20]	91.59	12.53	67.13	116.80
mu[21]	132.50	9.40	113.40	150.90
sigma.residual	38.64	7.35	26.95	55.48
sigma.with.line[1]	6926.00	3091.00	3212.00	14760.00
sigma.with.line[2]	1714.00	748.70	801.90	3603.00
sigma.with.line[3]	525.80	234.90	242.90	1113.00
sigma.with.line[4]	6719.00	2957.00	3113.00	14240.00
sigma.with.line[5]	3986.00	1766.00	1848.00	8491.00
sigma.with.line[6]	1126.00	503.30	519.30	2427.00
sigma.with.line[7]	11310.00	4958.00	5224.00	23820.00
sigma.with.line[8]	4590.00	2026.00	2131.00	9615.00
sigma.with.line[9]	963.30	427.40	441.20	2039.00
sigma.with.line[10]	5765.00	2759.00	2528.00	12760.00
sigma.with.line[11]	984.90	446.10	449.70	2121.00
sigma.with.line[12]	5870.00	2547.00	2731.00	12460.00
sigma.with.line[13]	2942.00	1307.00	1337.00	6272.00
sigma.with.line[14]	2932.00	1305.00	1341.00	6194.00
sigma.with.line[15]	1435.00	637.00	665.40	3023.00
sigma.with.line[16]	3253.00	1429.00	1505.00	6919.00
sigma.with.line[17]	1816.00	796.60	839.50	3817.00
sigma.with.line[18]	4120.00	1820.00	1882.00	8700.00
sigma.with.line[19]	9274.00	4025.00	4354.00	19330.00
sigma.with.line[20]	2728.00	1205.00	1251.00	5788.00
sigma.with.line[21]	1405.00	626.50	647.30	2980.00
(B) *E. coli*				
Theta	248.50	10.50	227.30	268.90
mu[1]	284.80	10.41	263.80	305.00
mu[2]	229.20	13.43	203.00	256.20
mu[3]	276.40	8.19	259.90	292.50
mu[4]	236.30	20.35	196.20	276.90
mu[5]	175.00	20.33	136.90	217.80
mu[6]	292.60	5.61	281.20	303.50
mu[7]	215.60	20.44	176.10	256.90
mu[8]	299.30	10.62	277.50	319.70
mu[9]	272.20	9.69	252.60	291.20
mu[10]	275.80	14.45	246.40	303.70
mu[11]	233.50	14.97	204.20	263.60
mu[12]	247.40	21.46	205.30	290.00
mu[13]	232.70	16.58	200.00	266.10
mu[14]	162.60	25.75	116.50	218.20
mu[15]	260.50	10.77	238.80	281.50
mu[16]	267.20	21.51	223.70	309.10
mu[17]	298.20	12.51	272.40	322.30
mu[18]	222.70	24.52	174.80	271.60
mu[19]	211.30	21.02	170.50	254.00
mu[20]	295.50	7.34	280.80	310.00
mu[21]	228.20	18.98	191.30	266.50
sigma.residual	43.77	8.67	29.53	63.69
sigma.with.line[1]	1701.00	764.30	786.80	3625.00
sigma.with.line[2]	3035.00	1325.00	1419.00	6382.00
sigma.with.line[3]	1055.00	468.40	488.40	2243.00
sigma.with.line[4]	8152.00	3576.00	3768.00	17210.00
sigma.with.line[5]	6476.00	3057.00	2878.00	14270.00
sigma.with.line[6]	473.60	211.30	218.70	1019.00
sigma.with.line[7]	7855.00	3446.00	3620.00	16670.00
sigma.with.line[8]	1742.00	789.10	798.60	3703.00
sigma.with.line[9]	1464.00	644.80	672.60	3086.00
sigma.with.line[10]	3529.00	1564.00	1622.00	7536.00
sigma.with.line[11]	3856.00	1702.00	1778.00	8202.00
sigma.with.line[12]	9578.00	4147.00	4461.00	20310.00
sigma.with.line[13]	4882.00	2148.00	2235.00	10360.00
sigma.with.line[14]	9887.00	4963.00	4191.00	22680.00
sigma.with.line[15]	1883.00	833.00	872.40	3973.00
sigma.with.line[16]	9301.00	4038.00	4330.00	19590.00
sigma.with.line[17]	2513.00	1115.00	1152.00	5317.00
sigma.with.line[18]	13180.00	5713.00	6076.00	27480.00
sigma.with.line[19]	8282.00	3619.00	3874.00	17360.00
sigma.with.line[20]	840.70	376.40	383.50	1794.00
sigma.with.line[21]	6765.00	2974.00	3131.00	14230.00

Data summary: Table A2 and A3.

**Table A2:** The mean and standard deviation (SD) lifetime fecundity of the lines for each block on the *B. pumilus* B215 food source

	Block 1		Block 2		Block 3	
			
Line	Mean	SD	Mean	SD	Mean	SD
1. N2	97.80	66.34	206.60	38.55	103.00	74.68
2. CB4853	64.00	18.84	126.20	27.09	66.60	31.62
3. JU1400	43.40	27.08	60.00	17.39	50.60	19.19
4. JU1401	119.00	68.72	112.80	106.94	116.80	65.37
5. JU1409	60.60	52.69	120.40	64.53	61.20	46.66
6. JU1410	144.00	26.33	142.20	43.94	140.20	27.07
7. JU1411	185.20	78.71	38.00	53.57	191.40	78.75
8. JU1416	110.40	74.82	107.60	54.52	112.20	73.01
9. JU1442	37.00	22.86	65.00	38.88	37.00	13.62
10. JU1494	202.60	37.03	160.00	110.48	195.40	32.92
11. JU262	25.60	21.20	50.40	37.67	20.20	19.85
12. JU319	140.80	58.15	37.60	48.72	142.60	55.67
13. JU345	156.40	32.00	83.60	53.22	150.40	30.11
14. JU362	59.80	39.28	52.20	71.50	69.00	44.02
15. JU393	83.60	15.31	75.80	56.89	80.00	28.79
16. JU400	155.60	23.68	63.20	41.78	154.60	21.50
17. MY1	126.80	19.73	106.40	68.43	120.00	11.42
18. MY16	66.40	67.70	34.40	44.53	56.60	71.41
19. MY2	8.20	5.54	155.60	96.01	3.20	7.16
20. PX174	109.00	23.35	58.60	72.68	104.60	22.41
21. PX179	113.40	23.86	171.20	29.30	120.00	16.40

**Table A3:** The mean and standard deviation (SD) lifetime fecundity of the lines for each block on the *E. coli* OP50 food source

	Block 1		Block 2		Block 3	
			
Line	Mean	SD	Mean	SD	Mean	SD
1. N2	325.40	32.54	278.80	5.50	257.40	31.63
2. CB4853	218.80	20.86	206.80	77.71	255.40	33.25
3. JU1400	295.00	29.78	254.60	27.28	283.00	21.64
4. JU1401	286.80	50.80	151.20	77.02	259.20	59.05
5. JU1409	212.20	20.10	153.00	99.19	107.00	33.08
6. JU1410	288.20	26.44	303.60	5.18	288.40	22.33
7. JU1411	224.60	51.93	186.40	78.71	207.40	117.48
8. JU1416	322.20	42.20	287.40	43.34	298.60	27.04
9. JU1442	284.40	17.98	276.40	24.61	260.00	55.49
10. JU1494	282.40	35.18	272.20	82.10	283.60	50.15
11. JU262	216.40	83.66	239.80	58.02	237.00	29.39
12. JU319	241.80	135.55	303.20	29.58	195.40	52.51
13. JU345	241.60	22.22	227.20	53.68	220.60	105.57
14. JU362	157.60	53.75	56.00	26.95	181.80	105.99
15. JU393	265.20	22.06	264.60	64.77	254.00	30.27
16. JU400	319.80	39.42	217.20	20.66	283.40	139.95
17. MY1	303.60	25.58	303.60	80.33	301.40	19.63
18. MY16	255.60	23.73	130.60	70.57	245.00	150.27
19. MY2	200.20	24.79	248.40	38.65	151.40	130.09
20. PX174	309.80	17.57	297.20	37.67	284.00	19.46
21. PX179	248.20	44.91	245.80	61.81	175.60	101.90

Supporting figures: Figure A1, A2 and A3.

## Appendix 3: WinBugs code

### Model 1

# model's likelihood

model{

for (i in 1:Nobs){

y[i]∼ dnorm(mu, tau)

}

#Priors

mu ∼ dnorm(theta,tau.residual)

tau∼ dgamma(0.001,0.001)

sigma <- sqrt(1/tau)

theta ∼ dnorm(0,1e10)

tau.residual ∼ dgamma(0.001,0.001)

sigma.residual <- sqrt(1/tau.residual)

}

### Model 2

# model's likelihood

model{

for (i in 1:Nobs){

mu[i] <- muS[line[i]]

y[i]∼ dnorm(mu[i], tau.among)

}

#Priors

for(j in 1:Nline){

muS[j]∼ dnorm(theta, tau.residual)

}

tau.among ∼ dgamma(0.001,0.001)

sigma.among <- sqrt(1/tau.among)

#Priors

theta ∼ dnorm(0,1)

tau.residual ∼ dgamma(0.1,0.1)

sigma.residual <- sqrt(1/tau.residual)

}

### Model 3

# model's likelihood

model{

for (i in 1:Nobs){

mu[i] <- muS[line[i]]

y[i]∼ dnorm(mu[i], tau.within.line[line[i]])

}

#Priors

for(j in 1:Nline){

muS[j]∼ dnorm(theta, tau.residual)

tau.within.line[j]∼ dgamma(0.001,0.001)

sigma.within.line[j]) <- (1/tau.within.line[j])

}

#Priors

theta ∼ dnorm(0,1.0E-10)

tau.residual ∼ dgamma(0.001,0.001)

sigma.residual <- sqrt(1/tau.residual)

}

### Model 4

#model's likelihood

model{

for (i in 1:Nobs){

mu[i] <- muS[line[i]] + alpha [exp1[i]]

y[i]∼ dnorm(mu[i], tau.within.line[line[i]])

}

#Priors

for(j in 1:Nline){

muS[j]∼ dnorm(theta, tau.residual)

tau.within.line[j]∼ dgamma(0.001,0.001)

sigma.within.line[j] <- sqrt(1/tau.within.line[j])

}

#Priors

theta ∼ dnorm(0,1.0E-10)

tau.residual ∼ dgamma(0.001,0.001)

sigma.residual <- sqrt(1/tau.residual)

#Priors

for(k in 1:Nexp){

alpha[k]∼ dnorm(0,1)

}

}

### Model 5

#model's likelihood

model{

for (i in 1:Nobs){

mu[i] <- muS[line[i]]+ alpha[exp1[i]]

y[i]∼ dnorm(mu[i], tau.among)

}

#Priors

for(j in 1:Nline){

muS[j]∼ dnorm(theta, tau.residual)

}

tau.among∼ dgamma(0.001,0.001)

sigma.among <- sqrt(1/tauS tau.among)

#Priors

theta ∼ dnorm(0,1)

tau.residual∼ dgamma(0.1,0.1)

sigma.residual <- sqrt(1/tau.residual)

#Priors

for(k in 1:Nexp){

alpha[k]∼ dnorm(0,1)

}

}

We wished to run three parallel chains. Below are the sets of initial values we used. For all the models, initial values were the same for both food sources: *E. coli* and *B. subtilis* data.

Model 1: list(tau =1, tau.residual =1)

list(tau =10, tau.residual =10)

list(tau =100, tau.residual =100)

Model 2: list(tau.residual =1, tau.among =1)

list(tau.residual =10, tau.among =10)

list(tau.residual =100, tau.among =100)

Model 3: list(tau.residual =1, tau.within.line =c(1, 1, 1, 1, 1, 1, 1, 1, 1, 1, 1, 1, 1, 1, 1, 1, 1, 1, 1, 1, 1))

list(tau.residual =10, tau.within.line =c(10, 10, 10, 10, 10, 10, 10, 10, 10, 10, 10, 10, 10, 10, 10, 10, 10, 10, 10, 10, 10))

list(tau.residual =100, tau.within.line =c(100, 100, 100, 100, 100, 100, 100, 100, 100, 100, 100, 100, 100, 100, 100, 100, 100, 100, 100, 100, 100))

Model 4: list(tau.residual =1, tau.within.line =c(1, 1, 1, 1, 1, 1, 1, 1, 1, 1, 1, 1, 1, 1, 1, 1, 1, 1, 1, 1, 1), alpha=c(0,0,0))

list(tau.residual =100, tau.within.line =c(10, 10, 10, 10, 10, 10, 10, 10, 10, 10, 10, 10, 10, 10, 10, 10, 10, 10, 10, 10, 10), alpha=c(0,0,0))

list(tau.residual =1000, tau.within.line =c(100, 100, 100, 100, 100, 100, 100, 100, 100, 100, 100, 100, 100, 100, 100, 100, 100, 100, 100, 100, 100), alpha=c(0,0,0))

Model 5: list(tau.among =1, tau.residual =1, alpha=c(0,0,0))

list(tau.among =10, tau.residual =10, alpha=c(0,0,0))

list(tau.among =100, tau.residual =100, alpha=c(0,0,0))

## References

[b1] Baer CF (2008). Quantifying the decanalizing effects of spontaneous mutations in rhabditid nematodes. Am. Nat.

[b2] Braendle C, Félix MA (2008). Plasticity and errors of a robust developmental system in different environments. Dev. Cell.

[b3] Braendle C, Baer CF, Félix MA (2010). Bias and evolution of the mutationally accessible phenotypic space in a developmental system. PLoS Genet.

[b4] Davidson CJ, Surette MG (2008). Individuality in bacteria. Annu. Rev. Genet.

[b5] Dubnau D, Losick R (2006). Bistability in bacteria. Molec. Micro.

[b6] Dunn RB, Fraser AS (1958). Selection for an invariant character- ‘vibrissae number’ -in the house mouse. Nature.

[b7] Félix MA, Duveau F (2012). Population dynamics and habitat sharing of natural populations of *Caenorhabditis elegans* and *C. briggsae*. BMC Biol.

[b8] Flatt T (2005). The evolutionary genetics of canalization. Q. Rev Biol.

[b9] Gibson G, Dworkin I (2004). Uncovering cryptic genetic variation. Nat. Rev. Genet.

[b10] Gillespie JH (1974). Natural selection for within-generation variance in offspring number. Genetics.

[b11] Hermisson J, Wagner GP (2004). The population genetic theory of hidden variation and genetic robustness. Genetics.

[b12] Hill WG, Zhang XS (2004). Effects on phenotypic variability of directional selection arising through genetic differences in residual variability. Genet. Res. Camb.

[b13] Hope IA (1999). C. elegans: A Practical Approach. The Practical Approach Series.

[b14] Horvitz HR, Sulston JE (1980). Isolation and genetic characterization of cell-lineage mutants of the nematode *Caenorhabditis elegans*. Genetics.

[b15] Lunn DJ, Thomas A, Best N, Spiegelhalter D (2000). WinBUGS – a Bayesian modelling framework: concepts, structure, and extensibility. Stat. Comput.

[b16] Maamar H, Raj A, Dubnau D (2007). Noise in gene expression determines cell fate in *Bacillus subtilis*. Science.

[b17] Rae R, Iatsenko I, Witte H, Sommer RJ (2010). A subset of naturally isolated *Bacillus* strains show extreme virulence to the free-living nematodes *Caenorhabditis elegans* and *Pristionchus pacificus*. Environ. Microbiol.

[b18] Raj A, Rifkin SA, Andersen E, van Oudenaarden A (2010). Variability in gene expression underlies incomplete penetrance. Nature.

[b19] Spiegelhalter DJ, Best NG, Carlin BP, van der Linde A (2002). Bayesian measures of model complexity and fit. J. Roy. Stat. Soc. B.

[b20] Starrfelt J, Kokko H (2012). Bet-hedging – a triple trade-off between means, variances and correlations. Biol. Rev.

[b21] Viney ME, Diaz A (2012). Phenotypic plasticity in nematodes: evolutionary and ecological significance. Worm.

[b22] Viney ME, Reece SE (2013). Adaptive noise. Proc. Roy. Soc. Lond. B.

[b23] Yang J, Loos RJ, Powell JE, Medland SE, Speliotes EK, Chasman DI (2012). FTO genotype is associated with phenotypic variability of body mass index. Nature.

